# Yeast to the rescue: *Meyerozyma guilliermondii* primes tomato vigor and resistance to Fusarium crown and root rot

**DOI:** 10.1080/15592324.2025.2596486

**Published:** 2025-12-05

**Authors:** Zayneb Kthiri, Maissa Ben Jabeur, Chahine Karmous, Walid Hamada

**Affiliations:** a Carthage University, Laboratory of Genetics and Cereal Breeding (LR14 AGR01), The National Agronomic Institute of Tunisia, Tunis, Tunisia; b Carthage University, Higher Institute of Preparatory Studies in Biology and Geology (ISEP BG), Soukra, Tunisia

**Keywords:** *Meyerozyma guilliermondii*, Fusarium crown and root rot, induced systemic resistance, biocontrol, Tomato

## Abstract

The use of biological control agents offers a sustainable alternative to chemical pesticides for managing soil-borne plant diseases. This study investigates the biocontrol potential of a newly isolated yeast strain, *Meyerozyma guilliermondii* INAT-MT731365, as a biotic elicitor to enhance growth and Fusarium crown and root rot (FCRR) resistance in hydroponically grown tomato plants. Tomato plants were treated with *M. guilliermondii* or left untreated as controls, then divided into two groups, one infected with *Fusarium oxysporum* f. sp. *radicis-lycopersici* (FORL) and one not infected. Physiological, biochemical, and molecular responses were monitored after treatment and inoculation. In the absence of the pathogen, *M. guilliermondii* treatment significantly enhanced plant growth and chlorophyll content. Concurrently, the yeast elicited a priming effect, characterized by low-level upregulation of PR1, *β*-1,3-glucanase and chitinase genes, downregulation of the P69G gene, and activation of defense enzymes such as peroxidase, chitinase, and *β*-1,3-glucanase, along with increased phenolic content and hydrogen peroxide accumulation, indicative of both SA- and JA/ET-mediated signalling induced systemic resistance (ISR). In control plants, FORL impaired plant defense with an early downregulation of *β*-1,3-glucanase and chitinase genes and stability in PR1 gene expression, followed by transient activation of peroxidase and chitinase and low activation of catalase, *β*-1,3-glucanase, and accumulation of phenolics. Upon FORL infection, treated plants exhibited strong upregulation of PR1, chitinase and *β*-1,3-glucanase genes mirrored by sustained increases in H₂O₂ and phenolic content and peroxidase, catalase, chitinase and *β*-1,3-glucanase activity. The simultaneous activation of both SA- and JA/ET-mediated signalling ISR resulted in a 61.8% reduction in FCRR severity and improved growth and photosynthetic traits. These findings highlight *M. guilliermondii* as a promising biocontrol agent that primes tomato plants for faster, stronger responses to soilborne pathogens while promoting growth under both healthy and stress conditions.

## 1 Introduction

Tomato (*Solanum lycopersicum*), a member of the Solanaceae family, is among the most widely cultivated and consumed vegetable crops worldwide. Its significance extends beyond culinary versatility to its vital role in global nutrition and economic development. As a high-value horticultural product, tomatoes consistently rank among the top vegetables globally, with annual output around 186 million tons in recent years.^1^ In Tunisia, tomato is a significant vegetable crop, cultivated on 22,000 hectares, accounting for 15% of the total area devoted to vegetable production, with an annual average production reaching 1.3 million tons, according to the latest published reports.^2^

However, many constraints affect productivity and quality of crops, and up to 40% of the world’s crops are lost to pests and diseases.^3^^,^^4^ Tomato productivity is threatened by over 200 diseases, with soilborne diseases being a major component.^5^ *Fusarium oxysporum* f. sp. *radicis-lycopersici* (FORL), the causal agent of Fusarium crown and root rot (FCRR), has been regarded as one of the most important threats to both field and greenhouse-grown tomatoes worldwide with 10–80% yield loss.^5-7^ The disease was first reported in Tunisia during the 2000–2001 cropping season and has caused plant losses of up to 90% in some geothermal greenhouses.^8^

The pathogen invades through root wounds or natural openings, colonizing the cortical and vascular tissues, particularly near the crown, leading to water transport disruption, stunted growth, and eventual plant decline. Early symptoms in seedlings include yellowing, cotyledon drop, and hypocotyl lesions, while mature plants exhibit leaf chlorosis, vascular browning, wilting, and reduced fruit quality. In severe cases, lesions extend into the root cortex and vascular system, with characteristic brown discoloration confined to the lower stem, distinguishing it from Fusarium wilt, where discoloration may extend up to 1 meter high.^9^^,^^10^ The long-term survival of the pathogen in soil as chlamydospores,^11^ along with its partial resistance to fungicides,^12^ makes management particularly challenging, underscoring the need for integrated and sustainable management strategies. These control challenges have prompted the search for effective and environmentally friendly alternatives, including the use of biological agents, nanotechnology, resistant cultivars, and biopolymers.^13^ The use of beneficial microorganisms for biological control, such as *Trichoderma* spp., *Pseudomonas* spp., *Rhizobium* spp and *Bacillus* spp have demonstrated efficacy in reducing FORL incidence by suppressing pathogen growth and activity in the rhizosphere.^14^^,^^15^ Biological control mechanisms include competition for space and nutrients, as well as antibiosis through the production of antimicrobial compounds such as hydrolytic enzymes, Volatile organic compounds, lipopeptides, and siderophores^13^^,^^15-17^).

In addition to direct antagonism, some beneficial microbes can enhance host plant defense by inducing systemic resistance, often triggering biochemical and molecular responses, involving the activation of signaling pathways associated with salicylic acid (SA), jasmonic acid (JA), and ethylene (ET)^15^.

Non-Saccharomyces yeasts are promising biocontrol agents (BCAs) due to their adaptability, broad pathogen suppression, and suitability for industrial use, acting through diverse mechanisms such as competition, antibiosis, mycoparasitism, and induction of host defense.^18^ One of the most frequently reported BCAs yeasts is *Meyerozyma guilliermondii* (formerly known as *Pichia guilliermondii*, Anamorph *Candida guilliermondii*), which is considered a nonconventional yeast with several unique biochemical and physiological properties. Its potential capabilities, such as industrial enzymes production, metabolites synthesis, and biocontrol activity, make it a promising species in the field of biotechnology.^19^ This species possesses biocontrol activity against postharvest diseases of fruits and vegetables^20^ through competition for nutrients, antagonism,^16^ and induction of defense-related enzymes and proteins. ^21^

Despite the demonstrated potential of these yeasts, the biocontrol and defense-inducing properties of *M. guilliermondii* against the soilborne pathogen FORL remain largely unexplored. In this context, we evaluated a recently isolated strain of *M. guilliermondii* for its ability to enhance tomato resistance to FCRR. This study aims to assess (i) the biocontrol efficacy of *M. guilliermondii* against FORL and (ii) its capacity to induce antioxidant enzymes and defense-related gene expression under infection conditions.

## 2 Materials and methods

### 2.1 Beneficial yeast culture and inoculation

**Figure 1.**
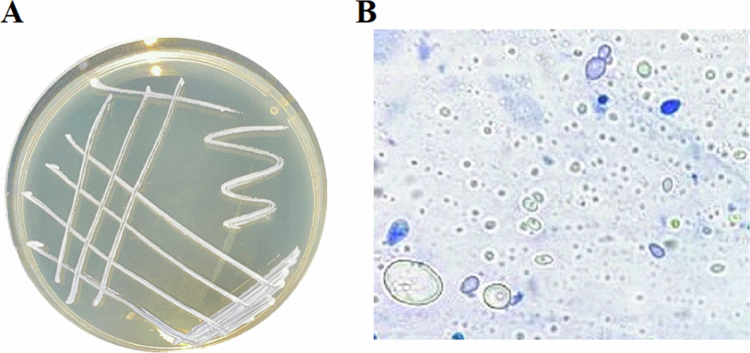
Macro and microscopic characteristics of *Meyerozyma guilliermondii* yeast strain INAT-MT731365 isolated from tomato stems (A) Colony morphology on PDA medium at 25 °C. (B) yeast cells.

The *Meyerozyma guilliermondii* yeast strain INAT-MT731365 (Figure 1) was isolated from tomato stems and characterized at the National Agronomic Institute of Tunisia (GeneBank accession number KU710283.1). Stem segments were surface-sterilized sequentially with 70% ethanol for 1 min, 2% NaOCl for 2 min, 70% ethanol for 30 s, and rinsed three times with sterile distilled water to remove epiphytic microorganisms. Sterilized segments were placed on potato dextrose agar (PDA) supplemented with chloramphenicol (100 mg L·¹) and incubated at 28 °C for 3 days. Emerging yeast colonies were purified by repeated streaking on PDA plates. Molecular identification of purified isolates was performed by PCR amplification and sequencing of the ITS rDNA region. A pure culture was maintained at 4 °C on PDA plates. A liquid culture was prepared by initiating yeast cells, from a single colony, and growing them in 50 ml of yeast extract dextrose (YD) medium in 250 ml flasks for 48 h at 28 °C. Cells were harvested by centrifugation at 5,000 rpm for 10 min at 4 °C, and resuspended in distilled water. The concentration of the cell suspension was then adjusted to 10^8^ cells ml·¹, corresponding to an optical density (OD) of 1.6 at 600 nm.

### 2.2 Pathogen strain and inoculation

**Figure 2.**
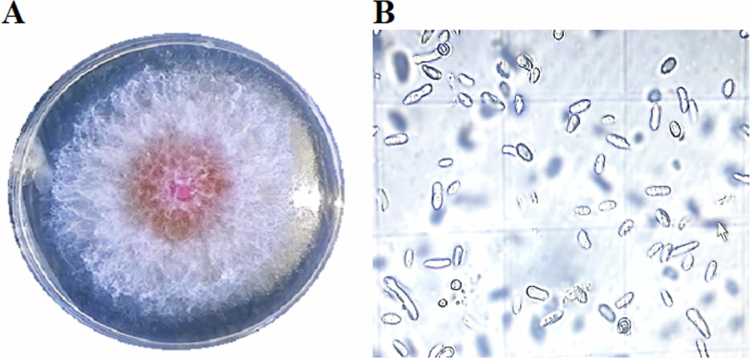
Macro and microscopic characteristics of *Fusarium oxysporum* f. sp. *radicis-lycopersici* (FORL) strain FORL-TOM24 isolated from infected roots of tomato (A) Colony morphology on PDA medium at 25 °C. (B) Micro and macro conidia.

*Fusarium oxysporum* f. sp. *radicis-lycopersici* (FORL) strain FORL-TOM24 (Figure 2) was obtained from infected tomato roots collected from greenhouses in Takelsa, Tunisia. The fungus was isolated and purified on potato dextrose agar medium (PDA) using the hyphal tip method. In detail, tomato roots showing root rot symptoms were cut into 1 cm and washed under running tap water. The obtained segments were immersed sequentially in 70% ethanol for 30 s, 1% NaOCl for 1 min, and rinsed three times with sterile distilled water. Sterilized root segments were placed on PDA supplemented with chloramphenicol (100 mg.ml^−1^) and incubated at 25 °C under 12 h light/dark photoperiod for 7 days. The culture was purified by transferring hyphal tips with typical *Fusarium*-like morphology (white-to-pink mycelial colonies, and morphology of macroconidia, microconidia and chlamydospores under the microscope) to fresh PDA plates. Pure cultures were stored in glycerol 20% at −20 °C.

A conidial suspension was prepared by adding 5 ml of 0.05% Tween 20 onto the surface of a 10-days old PDA plate, scraping the colonies with a glass rod, and collecting and filtering the suspension. The spore concentration was adjusted to 1 × 10^6^ spores ml·¹ using a hemocytometer.

### 2.3 Plant growth in hydroponic system and FORL infection

Tomato (*Solanum lycopersicum* L.) cv. ‘Rio Grande’ was used in this study. Seeds were surface sterilized for 1 min in 75% ethanol and then rinsed for 3 min with sterile distilled water to allow aseptic germination. The effect of *M. guilliermondii* on Fusarium crown and root rot was evaluated using a non-circulating hydroponic system. Fifteen-days old pre-germinated tomato seedlings, bearing 3 true leaves, were transplanted into plastic boxes containing 400 ml of Hoagland’s nutrient solution. The experiment was conducted in a growth chamber maintained at 22 ± 3 °C, 40–50% relative humidity, and a 16:8 h light/dark photoperiod with an average light irradiance of 350 µmol·m·²·s·¹.

**Figure 3.**
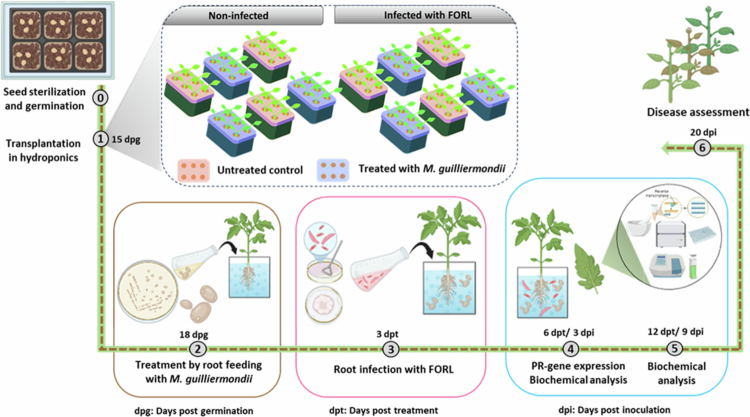
Schematic illustration of the completely randomized design and experiments used to assess the potential of *Meyerozyma guilliermondii* to control FORL.

After three days, plants were treated via root feeding by adding 4 ml of *M. guilliermondii* inoculum (10^8^ CFU ml·¹) to 400 ml of Hoagland’s nutrient solution, resulting in a final concentration of 10^6^ CFU ml·¹. FORL inoculation (10^6^ spores ml·¹) was applied three days after treatment (Figure 3). The following treatments were applied: (i) untreated and non-infected control, (ii) untreated and FORL-infected control, (iii) treated with *M. guilliermondii* only, and (iv) treated with *M. guilliermondii* and infected with FORL (Figure 3).

### 2.4 Quantification of defense-related gene expression

The expression of defense-related and antioxidative genes was analyzed by quantitative reverse transcription-polymerase chain reaction (qRT-PCR) at 3 dpi in leaves sampled from three replicates of infected and non-infected, and in treated and untreated plants. From each sample, 100 mg of tomato leaves were collected for RNA extraction. Total RNA was extracted using the FavorPrep Plant Total RNA Mini Kit (Favorgen Biotech, Taiwan) according to the manufacturer’s protocol. RNA samples were treated with RQ1 RNase-free DNase (M6101-RQ1; Promega, Madison, WI, USA) to remove contaminating DNA. RNA concentration and purity were assessed using a NanoDrop ND−2000 spectrophotometer.

First-strand cDNA was synthesized using the ImProm-II™ Reverse Transcription System (Promega, Madison, USA), following the manufacturer’s instructions. Reverse transcription conditions were: extension at 42 °C for 60 min, followed by enzyme inactivation at 70 °C for 15 min. The resulting cDNA was stored at −20 °C until use. Gene expression was quantified by qRT-PCR using specific primers for PR1, PR7, CHI3, and bGLUC genes, along with tubulin as a reference gene (Table 1). Real-time PCR was conducted using a LightCycler 480 system (Roche, USA) with the QuantiTect SYBR Green PCR Kit (Qiagen GmbH, Hilden, Germany) according to the manufacturer. The program used for qPCR was 1 cycle of denaturation for 3 min at 95 °C, followed by 40 cycles of: denaturation at 94 °C for 30 s, annealing at 60 °C for 30 s (Table 2), and extension at 72 °C for 30 s, then the reaction was kept at 4 °C. Following amplification, a melt curve analysis was performed by heating for 10 s at 95 °C, followed by 30 s at 60 °C, and then at 97 °C to assess the presence of oligonucleotide dimers. All reactions were run in technical triplicate for each biological replicate. No-template controls (NTC) were included to detect potential reagent contamination. Relative gene expression levels were calculated using the 2^−ΔΔCt^ method,^22^ with normalization to the reference gene tubulin.

**Table 1.** DNA sequences of real-time RT-PCR primers used for the amplification of selected PR protein genes in tomato.

Gene family	Specific class and Function	Primer sequence (5'-3')	Amplicon size (bp)	Accession
Tubulin	*β*-tubulin	TGGGATTTGCCCCACTAACC CAGGTAACGTCCATGACGGG	131	DQ205342
*PR1*	*PR1a* (P4), basic intracellular pathogenesis-related protein	AGTAGTCTGGCGCAACTCAG GTTCTCCAACCCAGTTGCCT	110	KY609511.1
*P69G*	PR7, acidic Subtilisin-like endoproteinase	TGGTGGATCTCTGAGACCTCTT GGCAGGCGAAGGACCATTAT	115	DQ157774
*Chitinase*	*PR3 (CHI3)*, Extracellular acidic endochitinase	TTGAATGTGGGATGGGTCCG CCCTGGGCGAAGTTCTTTTG	130	NM_001247475.2
Β−1,3-glucanase	*PR-2a,* Extracellular acidic *β*-1,3-glucanase	TCATGTGAAAGGAGGGGCAG CTTTGGCCTCTGGTCAGGTT	145	M80604.1

**Table 2.** Effect of *Meyerozyma guilliermondii* on shoot length, root length, biomass, and total chlorophyll content of tomato seedlings grown hydroponically and infected with FORL.

Traits	Shoot length (cm)		Root length (cm)		Biomass (g)		Total chlorophyll (mg. g^−1^ FW)	
Treatments	Non-infected	Infected	Non-infected	Infected	Non-infected	Infected	Non-infected	Infected
**Control**	15.36 ± 0.05	11.23 ± 1.00	12.63 ± 0.51	8.20 ± 0.78	2.61 ± 0.08	1.07 ± 0.20	26.38 ± 0.51	17.96 ± 2.62
***M. guilliermondii***	17.80 ± 0.55	14.13 ± 0.41	14.36 ± 0.70	13.43 ± 1.04	3.30 ± 0.10	2.04 ± 0.32	35.17 ± 0.38	24.09 ± 0.46

ANOVA	Sum squares with statistical significance							
**Treament (T)**	21.33***		36.40***		2.067***		166.77***	
**Infection (I)**	45.63***		21.60***		5.880***		285.07***	
**T x I**	0.16 ns		9.19**		0.059 ns		5.30 ns	

ns: non-significant, *: *P*< 0.05; **: *P* < 0.01; ***: *P* < 0.001.

### 2.5 Quantification of defense-related enzymes and metabolites

The activity of defense-related enzymes (catalase, peroxidase, chitinase, *β*-1,3-glucanase), as well as the content of phenolic compounds and H_2_O_2_ were evaluated at 3-, 6-, and 9-days post-inoculation (dpi), with each treatment sampled in triplicate (*n* = 3).

#### 2.5.1 Soluble phenolic compounds

Total phenolic content was estimated using the Folin–Ciocalteu method.^23^ Samples of 500 mg of fresh leaves were ground in 2 ml of 80% methanol at 4 °C and centrifuged at 1,000 rpm for 10 min. A 100 μl aliquot of the supernatant was added to the reaction mixture containing 50 μl of 20% sodium carbonate, 250 μl of Folin–Ciocalteu reagent (Sigma-Aldrich, Germany), and 1,75 mL of sterile distilled water. The mixture was incubated for 30 min at 40 °C, then cooled to room temperature. The absorbance was measured at 760 nm, and the phenolic content was expressed as mg·g·¹ fresh weight (FW) using catechol as the standard.

#### 2.5.2 Peroxidase activity

Peroxidase activity was determined using guaiacol as a substrate, measured at 470 nm.^24^ 500 mg of leaf tissue was homogenized in 5 ml of 50 mM potassium phosphate buffer (pH 5.5). The homogenate was centrifuged at 9000 rpm for 20 min at 4 °C, and the supernatant was collected as the crude enzyme extract. The reaction mixture contained 0.1 ml of crude enzyme, 2.9 ml of phosphate buffer (50 mM, pH 5.5), 1 ml of 0.6 M H₂O₂, and 1 ml of 50 mM guaiacol. Absorbance was recorded at 30-second intervals for 5 minutes at room temperature. Enzyme activity was expressed as Units·mg·¹ protein. Total protein content was determined using the Bradford assay,^25^ with absorbance measured at 595 nm. Bovine serum albumin (BSA) (Sigma, USA) used as the standard for the calibration curve.

#### 2.5.3 Catalase activity

Catalase (CAT) activity was determined following the method described by.^26^ Briefly, 100 mg of fresh leaf tissue was homogenized in 5 mL of 100 mM sodium phosphate buffer (pH 7.0) and thoroughly ground. The homogenate was centrifuged under the previously specified conditions to obtain the enzyme extract. For the assay, 0.2 mL of the enzyme extract was added to 3 mL of sodium phosphate buffer containing 0.2 mL of hydrogen peroxide (H₂O₂) as the substrate. The decomposition of H₂O₂ was monitored by measuring the decrease in absorbance at 240 nm using a spectrophotometer. One unit of CAT activity was defined as the amount of enzyme causing a decrease of 0.001 absorbance units per minute. The specific activity was expressed as units. g·¹ fresh weight.

#### 2.5.4 Chitinase activity

Chitinase activity was evaluated following Algam et al.^27^ Samples were collected at 3, 6, and 9 dpi with FORL. One gram of fresh leaf tissue was ground in liquid nitrogen, and the powder was dissolved in 5 ml of 0.1 M sodium citrate buffer (pH 5.0). The mixture was centrifuged at 10,000 rpm for 10 min at 4 °C, and the supernatant was collected. After 2 h of incubation at 37 °C, the reaction was stopped by centrifugation at 1,000 rpm for 3 min. An aliquot (0.3 ml) of the supernatant was added to 30 μl of 1 M potassium phosphate buffer (pH 7.1). *N*-acetylglucosamine (GlcNAc) release was quantified using internal GlcNAc standards. Enzyme activity was expressed as mM GlcNAc equivalents.min·¹.g·¹ FW.

#### 2.5.5 Β−1,3-glucanase activity

Fresh tomato leaves (1 g) were ground in liquid nitrogen and homogenized in 5 ml of 0.05 M sodium acetate buffer (pH 5.0). The extract was centrifuged at 10,000 rpm for 15 min at 4 °C, and the supernatant was collected. The reaction mixture included 62.5 μl of 20 mg·ml· ¹ laminarin (Sigma, USA) and 62.5 μl of the enzyme extract, incubated at 40 °C for 10 min. The reaction was stopped by adding 375 μl of dinitrosalicylic acid reagent, followed by boiling for 5 min. The resulting solution was diluted with 4.5 ml of distilled water, vortexed, and absorbance was measured at 500 nm. Enzyme activity was expressed as mmol.min·¹ .g·¹ FW.^28^ Final results were expressed in μmol Glc.min·¹.

#### 2.5.6 H_2_O_2_ content

Hydrogen peroxide (H₂O₂) content was measured as described by Noreen and Ashraf.^29^ 0.1 g of fresh shoot tissue was homogenized in 2 ml of 0.1% (w/v) trichloroacetic acid (TCA) and centrifuged at 12,000 rpm for 15 min. The assay mixture contained 0.5 ml of the supernatant, 0.5 ml of 10 mM phosphate buffer (pH 7.0), and 1 ml of 1 M potassium iodide (KI). Absorbance was recorded at 390 nm, and H₂O₂ concentration was expressed as μM.g·¹ FW.

### 2.6 Plant growth traits and disease assessment

At 9 dpi, root length, shoot length, dry biomass, and total chlorophyll content were measured.

Total chlorophyll content was determined according to the method of Witham et al.^30^ One hundred mg of fresh leaves were ground using a mortar and pestle in 20 ml of 80% acetone on ice (4 °C). The extract was centrifuged for 10 min at 1000 rpm at 4 °C. Absorbance of the supernatant was recorded at 645 and 663 nm using a UV–Vis spectrophotometer. Each treatment was replicated three times with three plants per replicate. The total chlorophyll (TC) content, expressed as mg. g^−1^ of FW, was calculated according to Bansal et al.^31^ as follows: $\mathrm{TC}\left(\mathrm{mg}.g^{-1}\mathrm{FW}\right)=10^{-3\;}\times [\left(20.2*A_{645}\right)+\left(8.02*A_{663}\right)]\times W\times V$ .

Where A_645_ and A_663_ are the absorbance values at 645 nm and 663 nm, respectively, V is the volume of the extract, and W is the fresh weight of the sample.

Fusarium crown and root rot severity was assessed on 24 plants visually at 20 dpi using a 0–5 rating scale adapted from Cerkauskas.^32^ Each plant was evaluated based on the degree of crown and root tissue discoloration and necrosis, as well as overall plant wilting symptoms. The scale was defined as follows: 0 = no symptoms; 1 = slight discoloration of crown or roots, no visible wilting; 2 = moderate discoloration of crown or roots with minor wilting; 3 = severe discoloration with moderate wilting and early signs of tissue collapse; 4 = extensive tissue necrosis and advanced wilting symptoms; 5 = complete decay of crown/root system or plant death.

### 2.7 Statistical analysis

All collected data were subjected to analysis of variance (ANOVA), and means were compared using Student’s *t*-test at *p* = 0.05. All statistical analyzes and figure generation were conducted using RStudio (version 1.1.463).

## 3 Results

### 3.1 *M. guilliermondii* modulates defense-related gene expression in tomato leaves under both infected and non-infected conditions

**Figure 4.**
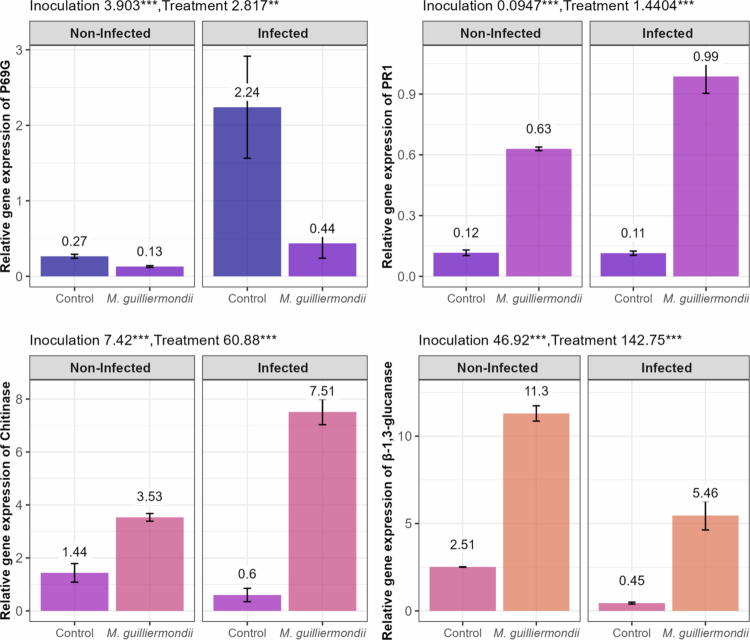
Relative expression of defense-related genes in tomato leaves at 3 days post-inoculation with FORL. Tomato plants were either untreated (control) or pretreated with *Meyerozyma guilliermondii*, and compared under non-infected and infected conditions. Expression levels were quantified by qRT-PCR and values represent mean ± SD of three biological replicates.

Statistical analysis via ANOVA confirmed that root treatment with *M. guilliermondii* and subsequent infection with FORL significantly altered the expression of key defense genes in leaves at 6 dpt (3 dpi) (Figure 4). Under non-infected conditions, *M. guilliermondii* treatment markedly upregulated the expression of PR1, *β*-1,3-glucanase, and chitinase genes by approximately 5.4-fold, 4.5-fold, and 2.4-fold, respectively, compared to untreated controls, suggesting priming of systemic resistance pathways in the absence of pathogen challenge. Conversely, P69G expression was slightly suppressed by approximately 2-fold in *M. guilliermondii*-treated, non-infected plants relative to controls.

In control plants, infection with FORL triggered a strong upregulation of P69G gene by approximately 8.4-fold. Conversely, *β*-1,3-glucanase and chitinase expressions decreased by nearly 5.6-fold and 2.3-fold, respectively in infected control plants relative to non-infected controls, suggesting that FORL may suppress certain defense pathways. Interestingly, PR1 transcripts remained low and barely changed (1.15-fold change increase in infected controls compared to non-infected plants).

Upon infection with FORL, *M. guilliermondii* treatment enhanced the expression of chitinase and *β*-1,3-glucanase genes by 12.5-fold and 12.2-fold, respectively, relative to infected controls, highlighting a strong activation of these defense enzymes. PR1 expression was also significantly upregulated (7.3-fold) in infected plants pretreated with *M. guilliermondii* compared to infected controls. Contrastingly, P69G gene expression showed a significant reduction by 5.2-fold in *M. guilliermondii*-treated infected plants compared to infected control plants. These results suggest that *M. guilliermondii* differentially modulates defense gene networks, potentially suppressing some pathogenesis-related proteins like P69G while strongly activating chitinase, *β*-1,3-glucanase, and PR1 pathways to enhance systemic resistance against FORL.

### 3.2 Defense-related enzymes and metabolites are activated by *M. guilliermondii* treatment under both infected and non-infected conditions

The analysis of variance (Appendix 1) revealed that *M. guilliermondii* treatment had a significant impact on tomato defense responses against FORL. Highly significant main effects were observed for time (T), infection (I), and treatment (Tr) were observed for most across nearly all defense-related biochemical markers, including peroxidase, chitinase, *β*-1,3-glucanase, hydrogen peroxide, and phenolic compounds. The most pronounced effects were detected for peroxidase, *β*-1,3-glucanase, and H₂O₂ (*p* < 0.001).

#### 3.2.1 Effects of *M. guilliermondii* under non-infected conditions: evidence of induced systemic resistance (ISR)

**Figure 5.**
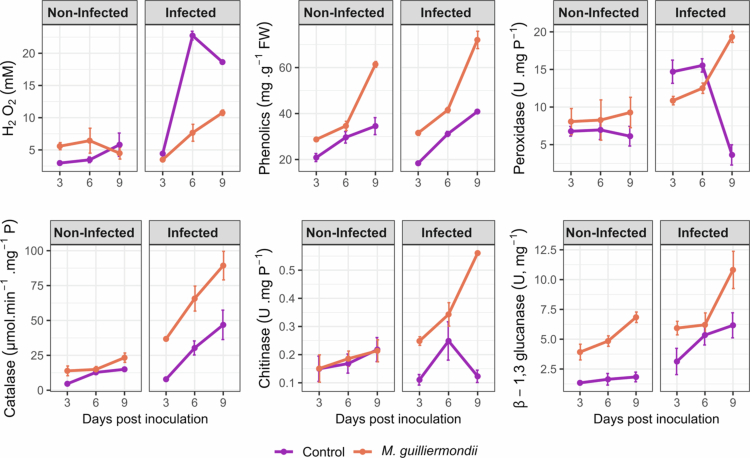
Time-course of defense-related biochemical responses in tomato leaves. Tomato plants were either untreated (control) or pretreated with *Meyerozyma guilliermondii*, and compared under non-infected and infected conditions. Values represent mean ± SD of three biological replicates.

In the absence of pathogen infection, *M. guilliermondii*-treated plants exhibited enhanced defense-related biochemical activity compared with untreated controls across all sampling times (Figure 5). Hydrogen peroxide (H₂O₂) levels increased progressively from 3 to 6 dpi (corresponding to 6 to 9 dpt), reaching values (6.43 mM at 6 dpi) nearly twice those in control plants (3.46 mM at 6 dpi), before slightly declining by 9 dpi (12 dpt). This moderate yet sustained H₂O₂ accumulation suggests that the yeast treatment induces a “primed” redox state typical of ISR. Phenolic content rose steadily throughout the experiment, showing a notable divergence from controls reaching 61.39 mg. g^−1^ FW at 9 dpi (12 dpt). Among enzymatic antioxidants, peroxidase and *β*-1,3-glucanase activities were notably elevated in treated plants, reaching and 9.27 and 6.83 U. mg P^−1^, respectively, at 9 dpi (12 dpt). These increases reflect the early activation of enzymatic defense related to cell wall strengthening and pathogen resistance. However, catalase activity exhibited a slight increase, while chitinase activity remained comparable to that of the control. Collectively, these results demonstrate that *M. guilliermondii* primes the tomato defense machinery depicted by a moderate accumulation of ROS, enhanced phenolic metabolism, and upregulation of key pathogenesis-related enzymes, hallmarks of ISR.

#### 3.2.2 Effects of *M. guilliermondii* under FORL infection: enhanced defense activation

Upon challenge with FORL, the activation of defense mechanisms was markedly stronger and more sustained in *M. guilliermondii*-treated plants compared to infected controls (Figure 5). Control plants exhibited a sharp oxidative burst, with H₂O₂ peaking at22.74 mM at 6 dpi before declining by 9 dpi. In contrast, treated plants maintained sustained and elevated H₂O₂ levels, suggesting a more controlled and prolonged defense response. Phenolic content, catalase, and *β*-1,3-glucanase in treated, infected plants increased steadily and was substantially higher than in controls at all time points. At 9 dpi, they reached the values of 72 mg. g^−1^ FW, 89 µmol. min^−1^.mg^−1^ *P*, and 10.81 U. mg P^−1^ respectively. These sustained elevations suggest coordinated activation of antioxidant defense and cell wall–degrading enzymes, which are known to restrict pathogen spread and reinforce resistance.

Notably, peroxidase and chitinase activity showed a significant decline in infected control plants by 9 dpi (3.63 and 0.123 U. mg P^−1^ respectively), whereas treated plants showed a strong and progressive upregulation of both enzymes across all time points, reaching 19.33 and 0.56 U. mg P^−1^ respectively at 9 dpi. This sustained activation of peroxidase and chitinase implies that yeast treatment enhances both oxidative and hydrolytic defense pathways, promoting structural and biochemical fortification against the pathogen. Overall, the combination of elevated ROS metabolism, phenolic accumulation, and increased activity of pathogenesis-related enzymes indicates that *M. guilliermondii* effectively primes tomato plants for faster and stronger defense activation under pathogen pressure.

### 3.3 *M. guilliermondii* promotes plant growth and chlorophyll content and mitigates FORL-induced damage

**Figure 6.**
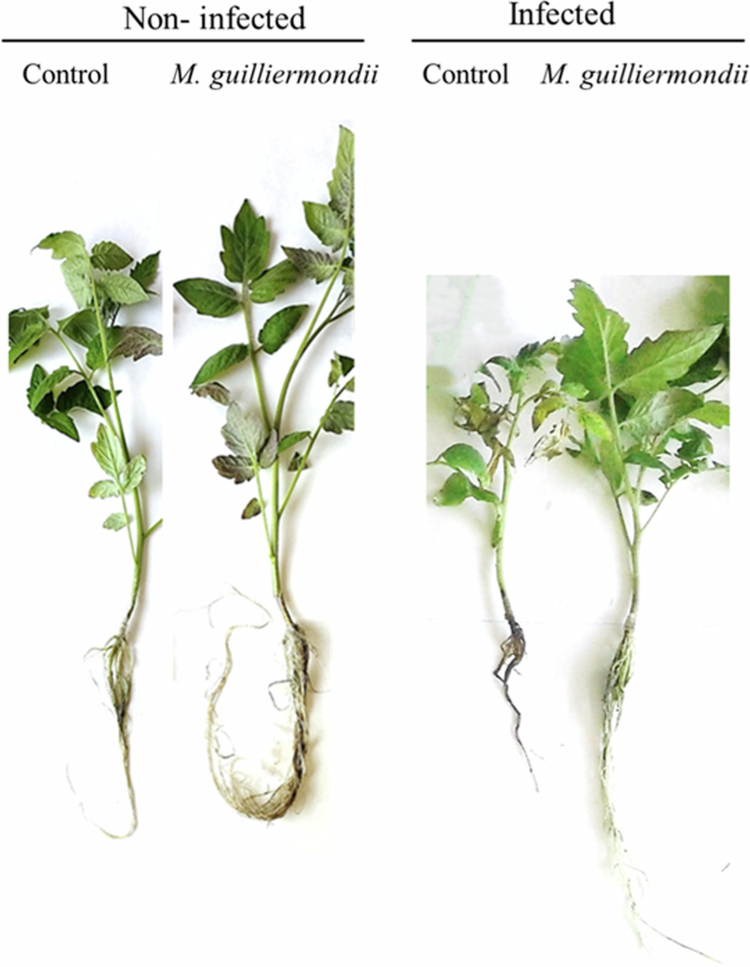
Effect of *Meyerozyma guilliermondii* on tomato plants at 20 days post inoculation with FORL. Representative tomato plants.

ANOVA results revealed highly significant effects (*p* < 0.001) of both treatment (T) and infection (I) on shoot length, root length, biomass, and total chlorophyll content, whereas the interaction term (T × I) was generally non-significant, indicating that *M. guilliermondii* exerted a consistent positive influence regardless of infection status. (Table 2, Figure 6). Under non-infected conditions, treatment with *M. guilliermondii* significantly enhanced all measured growth and physiological parameters in tomato seedlings compared to the untreated control. Shoot and root lengths were increased by approximately 15.8% and 13.7%, respectively, while biomass increased by about 26.4%, demonstrating a clear growth-promoting effect of the yeast under optimal conditions. Total chlorophyll content also rose markedly (by 33.3%), reflecting improved photosynthetic capacity and overall plant vigor.

In contrast, FORL infection significantly reduced all measured traits in control plants. Compared to the non-infected control, infected plants exhibited reductions of 26.9% in shoot length, 35.1% in root length, 59% in biomass, and 31.9% in total chlorophyll content (Table 2). These decreases underscore the severe inhibitory effects of FORL on plant growth and physiological function.

However, pretreatment with *M. guilliermondii* mitigated the negative impact of FORL, as evidenced by improved shoot and root lengths, biomass accumulation, and chlorophyll levels compared to infected controls (Table 2, Figure 6). The reduction rates for *M. guilliermondii*-treated plants were notably lower accounting for 20.6% in shoot length, 6.4% in root length, 38.18% in biomass, and 31.5% in chlorophyll content. This mitigation effect was particularly evident for root growth, which was almost maintained at non-infected levels.

### 3.4 *M. guilliermondii* reduces Fusarium crown rot and root rot disease severity

**Figure 7.**
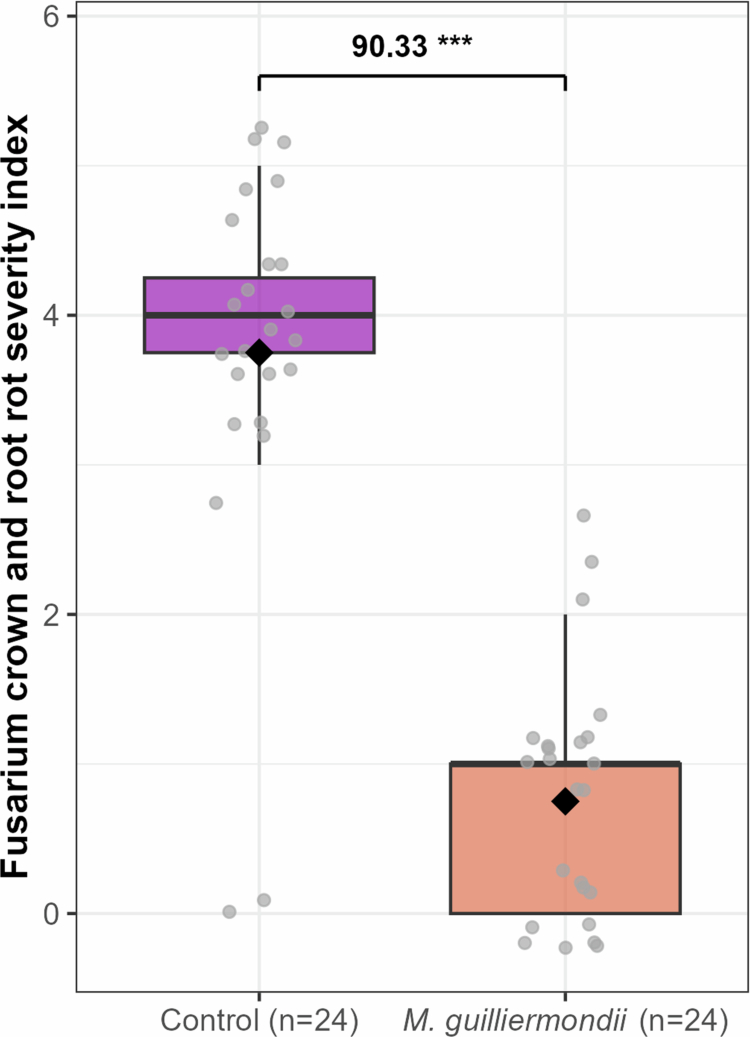
Boxplot illustrating the Fusarium crown root rot disease severity at 20 days post inoculation with FORL. Each treatment includes 24 independent experimental replicates (gray dots). Black Diamond-shaped markers indicate the mean, while box boundaries represent the interquartile range (25th–75th percentiles), with the horizontal line denoting the median. Whiskers extend to the minimum and maximum non-outlier values, and points beyond these are considered outliers. Asterisks indicate statistically significant differences (F-value and significance level shown).

Statistical analysis indicated a highly significant reduction of Fusarium crown and root rot severity (*p* < 0.001) in tomato plants pretreated with *M. guilliermondii* compared to untreated controls (Figure 7). As shown in Figure 7, the average disease severity index in control plants was 3.75, with median of 4.1 and an interquartile range (IQR) of about 3.8–4.4 on the 0–5 scale. These values corresponded with the observed severe root and crown necrosis, indicating high and consistent disease levels (Figure 7). In contrast, *M. guilliermondii*-treated plants showed a significantly lower average severity scores of 0.75, with lower median severity of approximately 0.5, and an IQR of 0.1–1.2. Several treated plants showed near-zero values, reflecting very mild or no visible symptoms. The distribution of individual data points confirms a consistent reduction in disease severity among treated replicates. These results demonstrate that *M. guilliermondii* treatment effectively suppressed FORL infection, substantially mitigating crown and root rot development. Overall, these findings highlight the potent potential of *M. guilliermondii* as a biocontrol agent against FORL in tomato plants.

## 4 Discussion

Plants acquire a range of mechanisms to shield themselves against regular attacks of potential pathogens and other stresses. In fact, the natural plant defense against various pathogens depends upon the early recognition of intra or extracellular components of pathogens.^33^ Plants treated with several beneficial microorganisms, including fungi and bacteria, can act as natural elicitors that promote the plant immune system through overexpression of defense-related enzymes and genes, increased accumulation of phenolic compounds, cell wall material synthesis, and overproduction of different signaling molecules.^34^^,^^35^ In the present study, we assessed a new isolated and characterized yeast strain of *M. guilliermondii* as a biotic elicitor on tomato plants grown in hydroponic system against the soilborne disease caused by FORL. This study demonstrates that *M. guilliermondii*, a yeast endophyte, can significantly promote tomato plant growth and improve physiological traits under both non-infected and FORL-infected conditions.

### 4.1 Impact of *M. guilliermondii* in the absence of pathogen: priming through ISR and growth promotion

**Figure 8.**
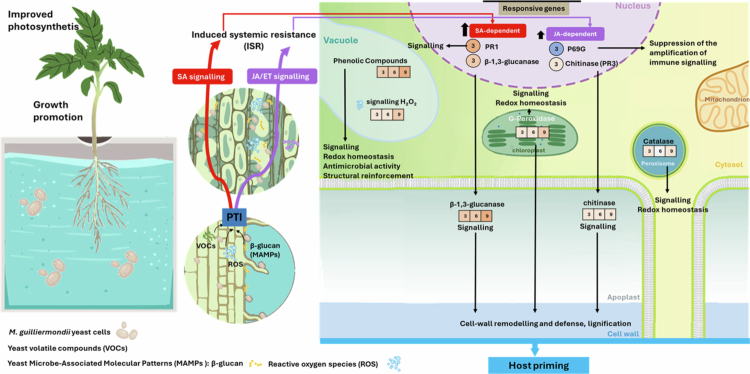
Putative systemic defense activation in tomato leaves induced by *Meyerozyma guilliermondii*: integration of SA and JA/ET signaling pathways within ISR.

Under non-infected condition, treatment with *M. guilliermondii* led to significant improvements in shoot and root growth, biomass, and chlorophyll content. This finding is consistent with studies reporting the ability of *M. guilliermondii* to promote plant growth.^36^ These effects likely stem from typical plant growth-promoting mechanisms such as enhanced nutrient uptake, hormonal modulation, or improved photosynthetic capacity.^37^^,^^38^ *M. guilliermondii* has been associated with increased indole−3- acetic acid (IAA) production and phosphate solubilization ability, further supporting its growth-promoting role^39,^ ^40^^,^^41^). However, the concurrent increase in defense-related genes expression and biochemical activities suggests more than just nutritional benefits. The low-level elevation of several defense-related markers after treatment with *M. guilliermondii* and in the absence of FORL indicates a priming of the plant immune system, consistent with ISR (Figure 8). Early basal transcriptional changes included upregulation of *β*-1,3-glucanase (PR2a) and chitinase (PR3) genes, coding for extracellular acidic *β*-1,3-glucanase and extracellular acidic endochitinase, respectively. *M. guilliermondii* is an endophyte that colonizes the rhizosphere.^42^ Taking into consideration that the cell wall of *M. guilliermondii* is composed predominantly of *β*-glucans and only minor amounts of chitin,^43^ *β*-glucan–rich cell wall fragments released from the yeast may act as microbe-associated molecular patterns (MAMPs) recognized by plant *β*-glucan receptors, thereby triggering stronger induction of *β*-1,3-glucanase gene expression compared with chitinase. Interestingly, the expression of PR1 gene, commonly associated with SA-dependent systemic acquired resistance (SAR),^44^ was also markedly upregulated, suggesting that SA accumulation is most likely induced by volatile organic compounds (VOCs) produced by *M. guilliermondii,* ^45^ which have been reported to trigger SA signaling.^46^ This pattern indicates that *M. guilliermondii* activates both SA- and JA/ET-associated defense signaling pathways, with stronger activation of SA-dependent pathway compared to JA/ET-dependent pathway. This supports the view that ISR triggered by beneficial microbes can involve coordinated activation of both SA- and JA/ET- dependent signaling branches.^47^ Meanwhile, the downregulation of P69G suggests a fine-tuning of proteolytic immune amplification, possibly to avoid hypersensitive or excessive defense responses. Subtilases such as P69 family members are known to mediate proteolytic cascades that amplify immune signaling during pathogen perception.^48^ Suppression of P69G by *M. guilliermondii* may therefore prevent excessive defense activation, facilitating a compatible plant–microbe interaction.

Biochemical analysis further supports the activation of ISR-related pathways and the promotion of growth in response to *M. guilliermondii*, characterized by: (i) enhanced accumulation of phenolic compounds as secondary metabolites involved in plant stress resistance through antioxidant activity, antimicrobial properties, cell wall reinforcement, and defense signaling^49^; (ii) basal activation of the enzymes peroxidase and chitinase, involved in antioxidative pathways, lignification, cell elongation and expansion and vascular development and potential antifungal action^50^^,^^51^; (iii) increased *β*-1,3-glucanase activity, facilitating and cell wall remodeling, cell expansion, tissue development, and early mobilization of defense mechanisms^52^; and (iv) a marginal increase of H₂O₂ and catalase, indicating a subtle but balanced oxidative response that did not result in stress symptoms. Collectively, these changes highlight the ability of *M. guilliermondii* to elicit a broad-spectrum primed state characteristic of ISR, integrating SA- and JA/ET-dependent signaling and enhancing defense readiness against both biotrophic and necrotrophic pathogens.

### 4.2 FORL infection: impairment of defense networks and plant physiology

**Figure 9.**
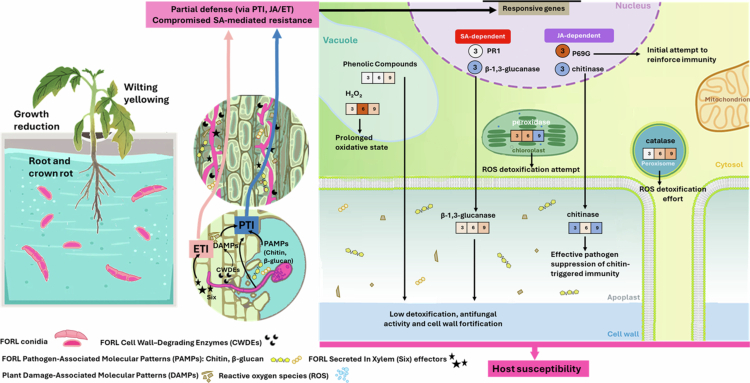
Putative defense impairment in tomato plants induced by *Fusarium oxysporum* f. sp. *radicis-lycopersici.*

The transcriptional and biochemical responses of tomato plants to FORL infection reveal a temporally dynamic yet ultimately ineffective defense activation (Figure 9). At 3 dpi, P69G expression was strongly induced, while PR2a (*β*-1,3-glucanase) and PR3 (chitinase) were repressed, and PR1 remained unresponsive, indicating an early but selective activation of defense signaling. The upregulation of P69G, a subtilase involved in proteolytic amplification of immune cascades,^48^ suggests that plants initially attempt to reinforce immune signaling upon pathogen recognition. However, the simultaneous downregulation of *β*-1,3-glucanase and chitinase transcripts indicates that FORL may actively suppress cell wall–degrading enzymes crucial for antifungal defense.^21^^,^^53^ The lack of PR1 induction further suggests that the SA pathway is bypassed or inhibited, consistent with reports that *F. oxysporum* manipulates the JA/ET signaling networks to promote host susceptibility.^54^

Biochemical analyzes supported this interpretation and revealed that the infection induced a pronounced but transient oxidative stress response in tomato plants, characterized by (i) an early surge in H₂O₂ levels; (ii) transiently increased activities of peroxidase and chitinase; and (ii) relatively lower activation of catalase, *β*-1,3-glucanase, and phenolic accumulation. These subdued and temporally restricted defense responses are consistent with FORL’s stealthy, vascular mode of infection. As a soil-borne pathogen, FORL enters through the roots and colonizes the xylem vessels, often evading early detection by host pattern recognition receptors and suppressing basal immunity.^55^^,^^56^ The insufficient defense activation in infected control plants suggests that FORL effectively dampens initial plant responses, enabling systemic colonization and disease development. The limited phenolic accumulation reflects an inadequate attempt to reinforce structural barriers and reduce oxidative damage. The combination of transient oxidative activation, delayed enzymatic responses, and suppressed antifungal gene expression supports previous reports indicating that FORL disrupts redox homeostasis and manipulates host signaling to facilitate infection.^54^^,^^57^

Consequently, these molecular and biochemical disruptions translated into severe physiological impairments, reflected by marked reductions in shoot and root length, biomass, and chlorophyll content. Such declines likely result from the cumulative effects of disrupted defense coordination, vascular colonization, and pathogen-induced interference with nutrient and water transport, consistent with the detrimental impact of *F. oxysporum* on plant growth and chlorophyll biosynthesis reported in other crops.^36^ Additionally, suppression of antioxidant defense, including decreased SA content and superoxide dismutase activity,^21^ likely exacerbates oxidative stress, contributing to pigment loss and cellular injury. Collectively, these findings demonstrate that FORL imposes multifaceted constraints on growth, photosynthetic efficiency, and redox homeostasis, enabling systemic infection and wilt symptom development in tomato plants.

### 4.3 Impact of *M. guilliermondii* in the presence of FORL: enhanced and sustained defense activation

**Figure 10.**
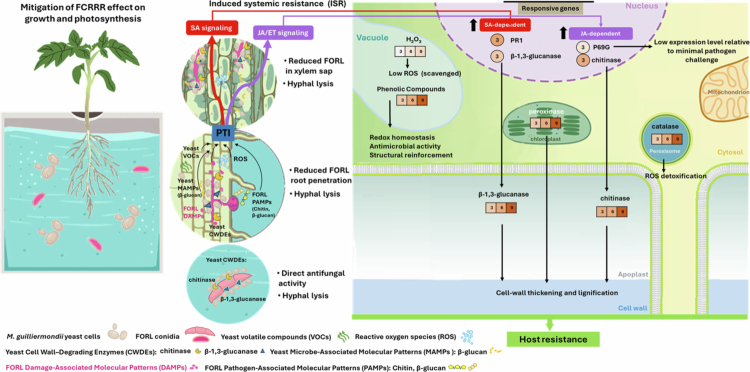
Putative synergistic direct and multilayered plant-mediated defense responses activated by *Meyerozyma guilliermondii* to confer host resistance against Fusarium Crown and Root Rot (FCRRR).

Under FORL infection, *M. guilliermondii*-treated plants exhibited significantly reduced disease severity compared to untreated controls. This yeast species has been reported as an effective bio-agent against the pathogens *Colletotrichum capsici* and *Ralstonia solanacearum.* ^58^ The observed maintenance of growth and photosynthetic parameters under pathogen pressure suggests that *M. guilliermondii* contributes to improved physiological resilience, likely through a combination of growth promotion and induced defense mechanisms. This protective effect is underpinned by robust and sustained defense responses at both the transcriptional and biochemical levels (Figure 10).

The transcriptional and biochemical responses of tomato plants to FORL infection were markedly enhanced by *M. guilliermondii* treatment, indicating a robust priming of host defense. At the molecular level, treated plants exhibited strong upregulation of chitinase and *β*-1,3-glucanase, enzymes often associated with JA/ET-dependent ISR and degradation of fungal cell walls,^21^^,^^59^ alongside increased PR1 expression, a hallmark of SA-dependent SAR. Considering the direct antifungal effect of *M. guilliermondii*, which antagonizes fungal pathogens through a lectin-like interaction resulting in strong adhesion to the fungal hyphae and pathogen cell lysis,^18^ the observed low activation of P69G suggests that direct antifungal activity of *M. guilliermondii* reduces the need for pathogen recognition–mediated amplification of basal immunity.

Biochemically, these transcriptional changes were mirrored by a sustained oxidative response, with elevated H₂O₂, peroxidase, catalase, and phenolic content throughout infection. The enhanced activities of chitinase and *β*-1,3-glucanase, together with the regulated ROS metabolism and antioxidant defense, indicate a coordinated strengthening of both structural and biochemical barriers. These observations suggest that *M. guilliermondii* primes the host for ISR, likely mediated by JA/ET signaling, while also supporting SA-dependent pathways for broader systemic resistance. The simultaneous activation of ISR- and SAR-associated markers highlights the capacity of this yeast to orchestrate a multi-layered defense, integrating hydrolytic, oxidative, and signaling components to effectively restrict pathogen colonization. Yu et al.^47^ summarized several studies reporting that ISR elicited by beneficial microbes involves the activation of both SA- and JA/ET-mediated signaling, highlighting the complex and diverse nature of the pathways underlying this defense response.

## 5 Conclusion

This study highlights the dual role of *M. guilliermondii* as both a plant growth promoter and an effective biotic elicitor of defense responses in tomato plants. Under non-infected conditions, *M. guilliermondii* enhanced growth parameters and photosynthetic capacity, and primed the plant immune system by inducing a low-level but strategic activation of defense-related enzymes and genes, indicative of ISR. In the presence of FORL, *M. guilliermondii* significantly mitigated disease severity by sustaining a strong biochemical and transcriptional defense response, mainly through activating JA/ET signaling, while also supporting SA-dependent pathways for broader systemic resistance. From a practical perspective, the low-nutrient-demanding endophyte *M. guilliermondii* represents a promising candidate for biotechnological applications as both biostimulant and biocontrol agent. It could be effectively applied through feasible techniques such as seed coating or priming, foliar sprays, and soil drenches using yeast-based formulations. These strategies have the potential to reduce reliance on chemical fungicides, lower production costs, and promote sustainable, environmentally friendly crop management. Overall, *M. guilliermondii* offers both biological and economic advantages, making it a valuable tool for integrated crop production.

## 6 Use of generative AI tools:

The authors used ChatGPT (OpenAI, GPT−4) to assist with improving the clarity and grammar of the manuscript text, generating code snippets for data visualization, and drafting parts of the methods section. All outputs were critically reviewed and edited by the authors to ensure accuracy and appropriateness. The authors take full responsibility for the content of the manuscript.

## Supplementary Material

Supplementary Material

## Data Availability

Data are available from the authors upon request.
