# Supplementary material for: Yeast to the rescue: Meyerozyma guilliermondii primes tomato vigor and resistance to Fusarium crown and root rot
Source: Plant Signal Behav. 2025 Dec 5;20(1):2596486. doi: 10.1080/15592324.2025.2596486 (PMC12688250; doi:10.1080/15592324.2025.2596486)
Supplement: Supplementary file 1 — Supplementary Material [file KPSB_A_2596486_SM8835.docx]

**Appendix 1.** Two-way ANOVA of the effects of *Meyerozyma guilliermondii* treatment, FORL infection, and time-course progression on defense-related enzymes and metabolites in tomato leaves.

| ANOVA | Peroxidase | Catalase | Phenolic compounds | Chitinase | Β-1,3-glucanase | H_2_O_2_ |
| --- | --- | --- | --- | --- | --- | --- |
| Time (T) | 9.10 | 4660*** | 4626*** | 0.079*** | 50.01*** | 277.2*** |
| Infection (I) | 241.73*** | 9244*** | 4. 165*** | 0.075*** | 73.78*** | 379.9*** |
| Treatment (Tr) | 53.13*** | 3997*** | 2230*** | 0.116*** | 91.17*** | 96.2*** |
| (T) x (I) | 10.45 **.** | 1949*** | 104*** | 0.017** | 8.08** | 209.4*** |
| (T) x (Tr) | 220.68*** | 84 | 805*** | 0.047*** | 12.80*** | 79.2*** |
| (I) x (Tr) | 2.37 | 1893*** | 57*** | 0.107*** | 1.54 | 198.3*** |
| (T) x (I) x (Tr) | 149.65*** | 99 | 1*** | 0.057*** | 2.71 | 87.9*** |

The sum square values with statistical significance are shown (ns: non-significant, *: p < 0.05; **: p < 0.01; ***: p < 0.001).
